# TCM-DS: a large language model for intelligent traditional Chinese medicine edible herbal formulas recommendations

**DOI:** 10.1186/s13020-025-01249-0

**Published:** 2025-11-17

**Authors:** Xuanfeng Li, Haining He, Guibin Lu, Peng Yue, Junying Chen, Zifeng Yang, Chitin Hon

**Affiliations:** 1 Respiratory Disease AI Laboratory in Epidemic Intelligence and Applications of Medical Big Data Instruments, Faculty of Innovation Engineering, Macau University of Science and Technology, Macau, China; 2 Institute of Systems Engineering, Macau University of Science and Technology, Macau, China; 3 Faculty of Medicine, Macau University of Science and Technology, Macau, China; 4 School of Software Engineering, South China University of Technology, Guangzhou, Guangdong China; 5 State Key Laboratory of Respiratory Disease, National Clinical Research Center for Respiratory Disease, Guangzhou Institute of Respiratory Health, The First Affiliated Hospital of Guangzhou Medical University, Guangzhou, Guangdong China

**Keywords:** Traditional Chinese medicine, Large language model, Domain adaptation, Edible herbal formula

## Abstract

**Background:**

The concept of *medicine and food homology* in traditional Chinese medicine (TCM) emphasized the dual role of certain material as both food and medicine, offering nutritional and therapeutic benefits. Edible herbal formulas, derived from this principle, are valuable for health management and chronic disease prevention.

**Methods:**

This study proposes a domain-specific prescription recommendation model enriched by TCM edible herbal formula knowledge called TCM-DS model. A dataset including symptoms, TCM constitutions, formulas and their corresponding ingredients was developed. DeepSeek R1 base model was fine-tuned utilizing Low-rank adaptation (LoRA) fine-tuning and a retrieval-augmented generation (RAG) module to increase recommendation accuracy. TCM-DS model was evaluated against general-purpose large language models.

**Results:**

The proposed TCM-DS model demonstrated superior performance, achieving a recommendation precision of 0.9924. Comparative experiments showed its robustness, with the highest precision scores for both forward and reverse symptom sequences compared with general-purpose large language models. A user-friendly platform was developed based on TCM-DS model, enabling automated constitution analysis and personalized formula recommendations.

**Conclusions:**

In conclusion, we proposed an intelligent TCM edible herbal formula recommendation model called TCM-DS. Its accompanying platform automated constitution identification and formula recommendation, advancing intelligent applications in TCM practice.

**Supplementary Information:**

The online version contains supplementary material available at 10.1186/s13020-025-01249-0.

## Background

The concept of *medicine and food homology* (MFH; Chinese: *药食同源*) in traditional Chinese medicine (TCM) dates back to ancient times, proposing that many materials function as both food and medicine, providing both nutritional and therapeutic properties [1, 2]. Originating from the *Huangdi’s Internal Classic* (Chinese: *《黄帝内经》*), this principle has become a cornerstone of TCM health preservation, particularly in the context of *preventive treatment of disease* (Chinese: *治未病*) [3]. Edible herbal formulas, composed of multiple medicinal and food homologous materials, exemplify this principle by synergistically combing dietary nutrition with therapeutic benefits. Distinct from conventional medications, edible herbal formulas exhibit minimal adverse effects and superior patient compliance, making them valuable for health management and chronic disease prevention [1]. Several medicinal and food homologous materials and their metabolites have demonstrated significant therapeutic potential in antioxidant and anti-aging effects, as well as the chronic disease prevention and treatment [4–7]. Conventional TCM prescription recommendations remain predominantly rely on practitioner expertise’s empirical knowledge, limiting their scalability in public health settings. This highlights the urgent need to develop an intelligent TCM prescription recommendation system.

Large language models (LLMs) have shown significant potential in TCM fields, leveraging their advanced natural language processing (NLP) capabilities and comprehensive knowledge bases. DeepSeek-R1 [8], a recently released cost-effective open-source LLM, offers unique advantages for medical research due to its local deployment capability which ensures data privacy. Furthermore, its open-source nature enhances model interpretability by making reasoning processes transparent to researchers.

The utilization of domain-specific corpus for model optimization has proven effective in enhancing the performance of general-purpose LLMs in specialized domains. In the field of TCM, several outstanding domain-specific LLMs have been developed through leveraging pre-training and fine-tuning approaches [9–12]. Beyond medical dialogue generation tasks, TCM prescription recommendation also represents a critical component in intelligent TCM diagnosis and treatment systems. Early studies on herb prescription generation mainly employed topic modeling [13, 14] and end-to-end models [15, 16]. The emergence of LLMs enables the creation of prescription generative model. The TCMBERT model proposed by Liu et al. [17] adopts a two-stage transfer learning framework that mimics the learning process of physicians, first acquiring medical knowledge through pretraining on TCM books and then fine-tuning with clinical records for prescription generation. Pu et al. [18] developed the RoKEPG model, which employs secondary pretraining on a corpus of Chinese herbal medicine and incorporates TCM knowledge via an attention mask matrix during fine-tuning. Zhuang et al. [19] further advanced this field with the TCM-LLaMA model which constructs a comprehensive TCM knowledge graph that integrates multidimensional information, including symptoms, tongue diagnosis, and pulse diagnosis, while enhancing performance through a novel synonym-based knowledge injection mechanism. Although these models have demonstrated strong performance, they primarily focus on generating traditional medicinal formulas and lack specialized frameworks for edible herbal formulas. Edible herbal formulas hold significant value in TCM's *preventive treatment of disease* theory, highlighting the need for dedicated recommendation systems.

Recent advances in DeepSeek-based LLMs have shown promising application in TCM or MFH field. Sha et al. [20] proposed Yaoshi-RAG, which combines DeepSeek-R1 with an uncertain knowledge graph and RAG to deliver personalized MFH dietary recommendations, demonstrating clear gains from KG-augmented reasoning. He et al. [21] developed OpenTCM, a GraphRAG-based TCM knowledge retrieval and diagnostic question answering (QA) system built from classical corpora, where DeepSeek-style Chinese LLMs assist KG construction and query understanding to improve ingredient retrieval and diagnostic support. Yuan et al. [22] conducted a nationwide survey reporting large-scale local deployments of DeepSeek-R1 in Chinese hospitals, underscoring the practicality of on-prem, privacy-preserving LLMs for clinical settings. Although these DeepSeek-based efforts demonstrate strong utility, they primarily focus on KG-augmented dietary advice or general QA task and lack a dedicated, constitution-aware framework for edible herbal formula recommendation. Our TCM-DS model addresses this gap by operationalizing a syndrome-first pipeline with LoRA fine-tuning and curated-database RAG.

In this study, we propose TCM-DS, an LLM specifically optimized for TCM edible herbal formula recommendations. By incorporating an external dataset of edible herbal formulas, TCM-DS model significantly enhances the recommendation accuracy, offering personalized edible herbal formula suggestions based on individual symptoms and TCM constitutions. Building upon the pre-trained DeepSeek-R1 base model, TCM-DS model incorporates two key technical innovations: (1) parameter-efficient fine-tuning via low-rank adaptation (LoRA), and (2) knowledge-enhanced generation through retrieval-augmented generation (RAG) module. This integrated architecture establishes domain-specific LLM for intelligent edible herbal formula recommendations.

## Methods

### Datasets

We systematically compiled a dataset on edible herbal formulas in accordance with the regulatory guidelines established by the National Health Commission (NHC) and the State Administration for Market Regulation (SAMR) of the People’s Republic of China. Based on the *Food Safety Law of the People’s Republic of China* and its implementing regulations, as well as the *Regulations Management of the Catalog of Substances Traditionally Used as Both Food and Medicinal Materials*, we conducted extensive searches using the key word “substances that are both food and Chinese medicinal materials” (Chinese: “既是食品又是中药材”) on the official website of the Department of Food Safety Standards, Risk Surveillance and Assessment [23]. As of August 31, 2024, we identified 426 substances that serve as both food and Chinese medicinal materials. Using this inventory, we performed targeted researches in classical TCM texts to retrieve edible herbal formulas composed of these dual-use substances along with their therapeutic effects. Ultimately, we collected 288 edible herbal formulas and 484 associated symptoms. Each formula was documented with its ingredients, therapeutic effects, and target population. To ensure accuracy and clinical relevance, licensed TCM practitioners classified and organized the formulas based on their therapeutic effects and suitability for different constitutional types. According to the nine-point method of Qi Wang [24], constitutions can be classified as balanced constitution, yang deficiency constitution, yin deficiency constitution, qi deficiency constitution, qi stagnation constitution, phlegm-dampness constitution, dampness heat constitution, blood stasis constitution, inherited special constitution.

Consequently, we compiled a database (Samples are shown in Table S1) encompassing 288 edible herbal formulas and their corresponding therapeutic effect, 426 substances that serve as both food and Chinese medicinal materials, 484 associated symptoms, and 9 constitutions. This dataset provides a systematically organized resource for research on TCM edible herbal formulas, bridging historical prescriptions with modern regulatory frameworks while preserving the integrity of classical Chinese medicine knowledge.

### TCM-DS model

**Fig. 1:**
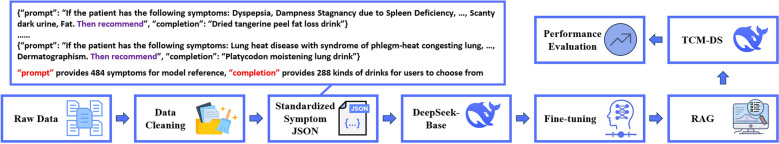
Overall workflow of TCM-DS model

TCM-DS was developed and optimized based on the Deepseek-LLM-7B base model, employing LoRA [25] fine-tune and RAG module to achieve precise personalized recommendations of edible herbal formulas. The workflow is shown in Fig. 1.

#### Data cleaning

The raw symptom data underwent a comprehensive cleaning pipeline to ensure data quality and consistency. First, term standardization was performed to colloquial expressions into standardized TCM terminology (e.g., translating “get pimples” [Chinese: 长痘痘] to “susceptible to scabies or acne” [Chinese: 易生疥疮或痤疮]). Next, duplicate symptoms were identified and merged. Subsequently, category mapping was conducted to organize related symptoms into predefined clinical classification. Finally, irrelevant or non-clinical data were filtered out. The cleaned data were then transformed into a structured a JSON format, optimized for downstream modeling applications.

#### Construction of prompt-completion dataset

The structured symptom data were formatted into *prompt*-*completion* pairs, where *prompt* provides a symptom combination with instructional phrasing (e.g., “If the patient presents with [symptom list]. Then recommend”), and the *completion* provides the matching edible herbal formula name. (Samples are shown in Fig. 1 and Table S2). The prompt templates were developed with guidance from licensed TCM practitioners, followed a symptom combination-recommended formula framework that adheres to the TCM principle of *syndrome differentiation and treatment* (Chinese: *辨证论治*). The model operated under the matching maximization principle, requiring the model to provide recommendations in prioritized order. An error-handling mechanism was implemented to return "NA" when encountering unidentifiable patterns, while output standardization protocols enforce structured formatting by eliminating punctuation and constraining responses to predetermined parameters to ensure consistent, machine-processable results.

The TCM-DS pipeline achieved syndrome differentiation and treatment recommendation by first standardizing raw symptom descriptions and grouping them into syndrome candidates using expert-defined mapping tables. In parallel, the system inferred the user’s constitution through a structured questionnaire based on Qi Wang’s nine-constitution method [24], with each formula in the database pre-labeled by licensed practitioners for applicable constitutions. Training examples were constructed as prompt–completion pairs that combined symptom clusters and constitution labels with their corresponding formula recommendations. During inference, the model jointly analyzed symptoms and constitution, retrieved relevant formula records via RAG, and employed the LoRA-fine-tuned module to generate a prioritized list of formulas. This layered design—symptom normalization, syndrome/constitution inference, evidence retrieval, and conditional generation—ensures the model emulates the clinical reasoning process of synthesizing symptoms into a syndrome pattern before selecting treatment, rather than directly matching herbs to isolated symptoms.

Licensed TCM practitioners curated a controlled vocabulary that linked each symptom to one or more syndrome patterns with designated primary and secondary weights. To avoid bias toward longer symptom lists, we used a length-normalized syndrome score:

$$\text{Score}(S)=\frac{\sum_{{x}_{i}\in X} w({x}_{i},S)}{\sum_{{x}_{i}\in X} {w}_{max}({x}_{i})}\in [\text{0,1}]$$

where *w(symptom, S)* is the practitioner-assigned weight of the symptom toward syndrome S (primary > secondary) and *w_max(symptom)* is the maximum possible weight that the symptom can contribute toward any syndrome. Syndromes with scores above a development-set threshold were retained (top K); otherwise, the output was NA.

A structured questionnaire (as described in [24]) provided a probability distribution over the nine constitutions. Syndrome and constitution were combined multiplicatively with a small smoothing term:

$${\text{Score}}^{*}(S,C)=\text{Score}(S)\left(\begin{array}{c}\varepsilon +P(C\mid X)\end{array}\right)$$

where *P(C | symptoms)* is the estimated probability of constitution C from the questionnaire, and *epsilon* is a small positive constant (e.g., 1e-3 to 1e-2). Top K (S, C) pairs above a fixed threshold proceeded to retrieval and generation; if none, the output was NA.

Prompts followed an instruction-then-structure template that required syndrome-first reasoning; when no reliable match was found, the output was set to “NA”. Completions reported the canonical syndrome label (SYNDROME), the inferred constitution (CONSTITUTION), and an ordered list of formula identifiers (TOPN), or a single “NA”.

#### Fine-tuning

**Fig. 2:**
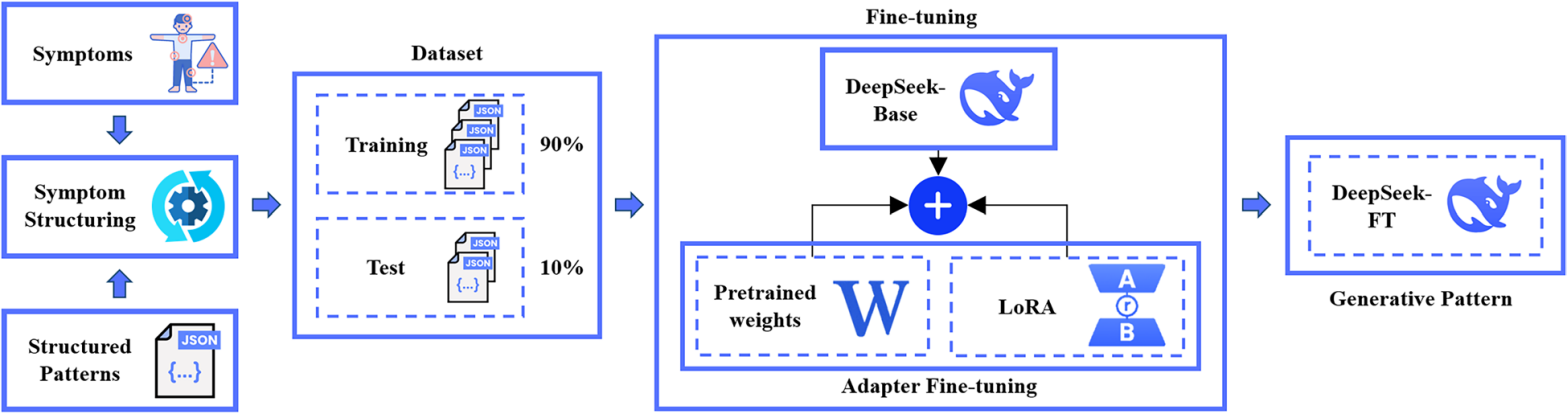
Adapter-based fine-tuning framework

The pretrained LLM DeepSeek-Base was used as base model, which was fine-tuned to construct the generative model DeepSeek-FT for edible herbal formula recommendations. The model was trained to learn the mapping between structured symptom prompt and target formulas, enabling conditional generation of personalized recommendations. The workflow is shown in Fig. 2. To address the computational inefficiency of full fine-tuning and mitigate overfitting risks, we adopted parameter-efficient LoRA. This method integrated trainable low-rank adaptation modules into the original model, substantially reducing the number of parameters requiring updates while maintaining performance. The data set was randomly split into training set and test set at a 9:1 ratio. The fine-tuning process involved loading the pretrained DeepSeek-Base model and applying LoRA-based adaptation. Only the low-rank matrices within the LoRA module were updated through gradient descent, keeping the original model parameters frozen.

#### Retrieval-augmented generation (RAG) module

**Fig. 3:**
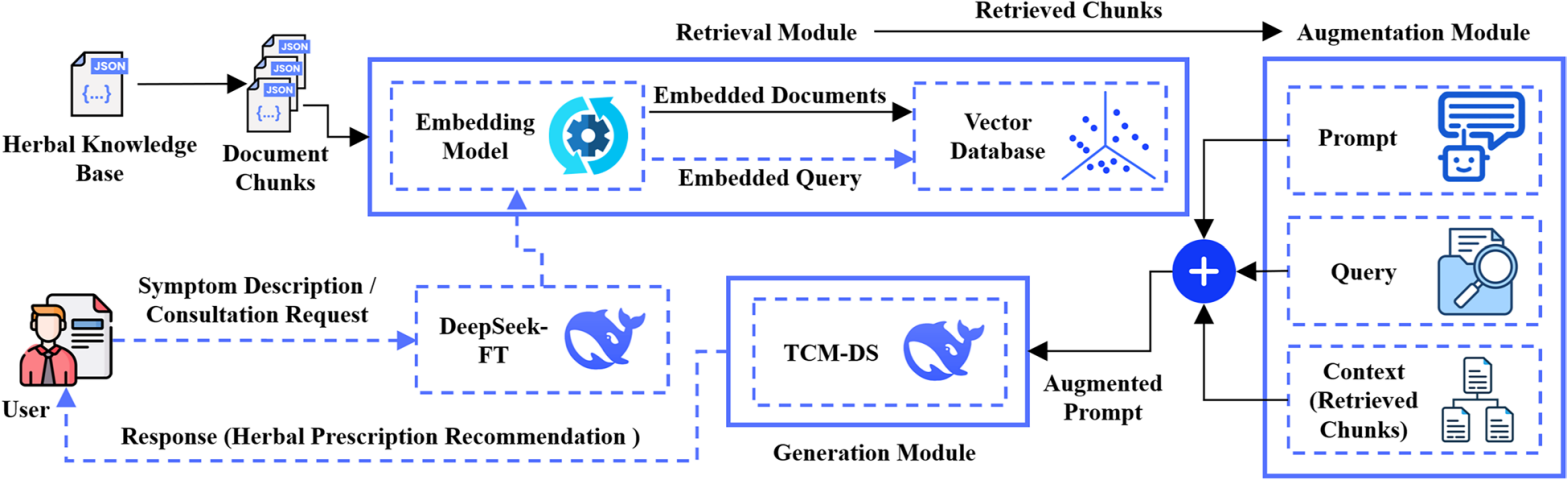
RAG-enhanced inference framework for edible herbal formula recommendation in TCM-DS model

RAG framework was used to extend the DeepSeek-FT model, constructing the TCM-DS model to enhance the contextual relevance of generated responses. By incorporating relevant content from TCM edible herbal formula dataset, this framework could improve the rationality and interpretability of recommendations. The workflow is shown in Fig. 3. The implementation involved preprocessing dataset into structured JSON files, which were then segmented into retrievable document chunks. These chunks were encoded using a embedding model and stored in a vector database. When a user submitted a query, the system generated a query vector and retrieved the most relevant document chunks. Standardized symptoms, together with the inferred constitution, jointly defined the query. Candidate formulas were ranked by a transparent composite score that accounted for indication overlap, constitution compatibility, symptom coverage, and contraindication penalties, with the weights tuned on the development set (see Table S3).

Specifically, we used a linear, interpretable composite score:

$$R(F\mid S,C,X)=a\text{Ind}(S,F)+b\text{Compat}(C,F)+c\text{Cov}(X,F)-d\text{Contra}(F)$$

where *Ind(S, F)* denotes the overlap between the inferred syndrome and the formula’s documented indications (e.g., Jaccard or proportion match); *Compat(C, F)* denotes the compatibility between the inferred constitution and the formula’s target population (1 for fully compatible, fractional for partial); *Cov(symptoms, F)* denotes the proportion of reported symptoms covered by the formula’s documented effects; *Contra(F)* denotes the penalty for contraindications or conflicts (0 when none, closer to 1 for strong conflicts); and *a, b, c, d* are non-negative weights satisfying *a* + *b* + *c* + *d* = *1*, tuned on a development set and fixed for evaluation.

The top-M evidence snippets were appended to the prompt as external knowledge. These retrieved chunks were integrated with the original query through an enhancement module to form an augmented prompt containing external knowledge. Finally, the TCM-DS model generated personalized edible herbal formula recommendations based on the augmented prompt, ensuring tripartite alignment among symptom descriptions, constitutional characteristics, and professional TCM knowledge, thereby completing the closed-loop question-answering recommendation system.

Constitution analysis was implemented through a structured questionnaire derived from the Classification and Determination of Constitution in TCM (ZYYXH/T 157–2009), covering the nine types described by Qi Wang (balanced, qi-deficiency, yang-deficiency, yin-deficiency, phlegm-dampness, damp-heat, blood stasis, qi-stagnation, and inherited special constitution) [24]. User responses were automatically scored using the standard algorithm, which accounts for positive and negative items, threshold cutoffs, and mutual exclusivity rules, ensuring accurate classification.

Each edible herbal formula in the curated database was pre-labeled with one or more suitable constitution types by licensed TCM practitioners. Once a user’s constitution is inferred, this label is integrated as an intermediate representation in the recommendation pipeline, ensuring that formula selection reflects the principle of *treatment tailored to the individual* (Chinese: *因人制宜*). For example, when two users both present with dyspepsia and bloating, the system differentiates between yin-deficiency and phlegm-dampness constitutions: the former leads to recommending Poria cocos appetizing drink, while the latter corresponds to dried tangerine peel fat loss drink. This mechanism reflects the principle of *different treatments for the same disease* (Chinese: *同病异治*). By combining structured questionnaire–based constitution analysis with formula–constitution labeling, TCM-DS ensures that recommendations are not only symptom-driven but also constitution-aware.

### Evaluation

#### Evaluation metrics

To validate the accuracy and reliability of TCM-DS model in recommending edible herbal formulas, we evaluated the prediction results using *Precision*. Precision: the proportion of formulas recommended by the model that is actually consistent with the symptoms and needs of the patient, calculated as:

$$Precision=\frac{TP}{TP+FP}$$

where *TP* refers to the numbers of formulas correctly recommended by the model, and *FP* refers to the number of formulas incorrectly recommended by the model. A high precision score indicates that most of the formulas recommended by the model are relevant and accurate.

#### Comparison with other LLMs

Comparative experiments between proposed TCM-DS model and mainstream general-purpose LLMs, including Qwen-Plus-latest, ERNIE-4.0–8.0.0.0 K, ChatGPT-4o-latest, and DeepSeek-R1-671B-chat, to evaluate their performance differences in edible herbal formula recommendation tasks.

During query processing, the input symptom descriptions were transformed into high-dimensional vector representation through the embedding layer. This vectorization preserved the semantic feature of the original text while enabling efficient retrieval in the vector database. When symptoms were arranged in forward order (e.g., dyspepsia, spleen deficiency with dampness exuberance, phlegm heat with cough, abdominal distension, abdominal obesity, scanty dark urine, obesity), the generated semantic vectors reflected the intrinsic relationship among symptoms in the vector space. Conversely, reverse ordering (e.g., obesity, scanty dark urine, abdominal obesity, abdominal distension, phlegm heat with cough, spleen deficiency with dampness exuberance, dyspepsia) produced distinct vector representations that alter semantic association patterns. These differences subsequently affected retrieval results from the vector database and ultimately influenced the generated formula recommendations. Accordingly, we testes both forward and reverse input sequences across all compared LLMs and the proposed TCM-DS model to evaluate their performance variations in formula recommendation.

### Experimental settings

In this study, we employ LoRA to enable parameter-efficient fine-tuning of a large language model (Table S3). The base model is loaded with torch_dtype = torch.bfloat16 and device_map = "auto" to support mixed-precision training and automatic device placement across multiple GPUs, thereby optimizing memory usage.

For LoRA configuration, the task type is set to causal language modeling (CAUSAL_LM). We inject low-rank adaptation modules into key attention layers, specifically q_proj, v_proj, and down_proj. The rank (r) is set to 8, the scaling factor (lora_alpha) is 32, and the dropout rate is 0.01 to mitigate overfitting. The bias term is not trained (bias = "none"), ensuring minimal modification to the original model parameters.

Regarding training hyperparameters, the per-device training batch size is set to 4, and gradient accumulation steps is configured to 2 to simulate a larger effective batch size, which improves optimization stability. The learning rate is set to 2e-5, and training is conducted for 3 epochs. We adopt the AdamW optimizer (PyTorch implementation) and enable fp16 mixed-precision training to further reduce memory consumption. Logging is performed every 50 steps, and model checkpoints are saved every 500 steps. Training metrics are reported to TensorBoard for real-time monitoring. Additionally, we apply 100 warm-up steps to stabilize the early training stage.

## Results

### Selection and evaluation of base model

During the selection process of the base model, we comprehensively evaluated inference efficiency, training costs, and available hardware resources, ultimately limiting candidate models to the parameter range of 3B to 15B to achieve optimal balance between performance and computational consumption. Following these criteria, we selected five mainstream open-source models for experimental evaluation: LLaMA2-8B[26], ChatGLM3-6B [27], Mistral-7B, Qwen2.5-7B [28], and Deepseek-LLM-7B [29]. Comparative tests conducted in a standardized experimental environment demonstrated that Deepseek-LLM-7B exhibited superior performance with precision of 0.3848 (Table S4). Furthermore, the model’s MIT license permits commercial applications and secondary development, while its native support for LoRA fine-tuning significantly reduces technical barriers for domain adaptation. Based on the evaluation results and technical advantages, we ultimately selected Deepseek-LLM-7B as the base model for subsequent fine-tuning and RAG-based model development.

### Ablation study

To validate the effectiveness of LoRA fine-tuning and RAG methods, ablation experiments were conducted on the testing set. Table 1 shows the results. The base model without any modifications achieved a recommendation precision of 0.3848. When fine-tuning with LoRA alone, the precision significantly improved to 0.6711. Implementation of only the RAG module resulted in an precision of 0.9082. The complete TCM-DS model incorporating both LoRA fine-tuning and RAG module achieved optimal performance with a recommendation accuracy of 0.9924, demonstrating the complementary benefits of combining these two approaches for domain-specific adaptation in TCM.

### Overall performance of TCM-DS model

To evaluate the effectiveness of the proposed model, we compared the performance of TCM-DS model with several general-purpose LLMs. As shown in Table 2, the evaluation results on the test set demonstrate that TCM-DS model achieved optimal performance under both forward and reverse order input conditions, with precision of 0.9924 and 0.9702, respectively. DeepSeek-R1-671B-chat exhibited the second-best performance, maintaining accuracy above 0.9000 for both input conditions (Table 2). Notably, ChatGPT-4o-latest achieved an accuracy of 0.9854 with forward order inputs but showed decreased performance with reverse order inputs. Conversely, ERNIE-4.0–8.0.0.0 K demonstrated higher accuracy with reverse order inputs.

**Table 1:** Performance comparison in ablation study

Model	Precision
*w/o* LoRA&RAG	0.3848
*w/o* LoRA	0.9082
*w/o* RAG	0.6711
TCM-DS	**0.9924**

‘*w/o*’ represents ‘without’. LoRA: Low-rank adaptation, RAG: Retrieval-augmented generation

**Table 2:** Performance comparison between TCM-DS model and several universal large language models

Model	Precision	
	Forward order	Reverse order
Qwen-Plus-latest	0.7615	0.8322
ERNIE-4.0–8.0.0.0 K	0.8344	0.9597
ChatGPT-4o-latest	0.9854	0.8945
DeepSeek-R1-671B-chat	0.9738	0.9301
**TCM-DS (ours)**	**0.9924**	**0.9702**

### Case study

**Fig. 4:**
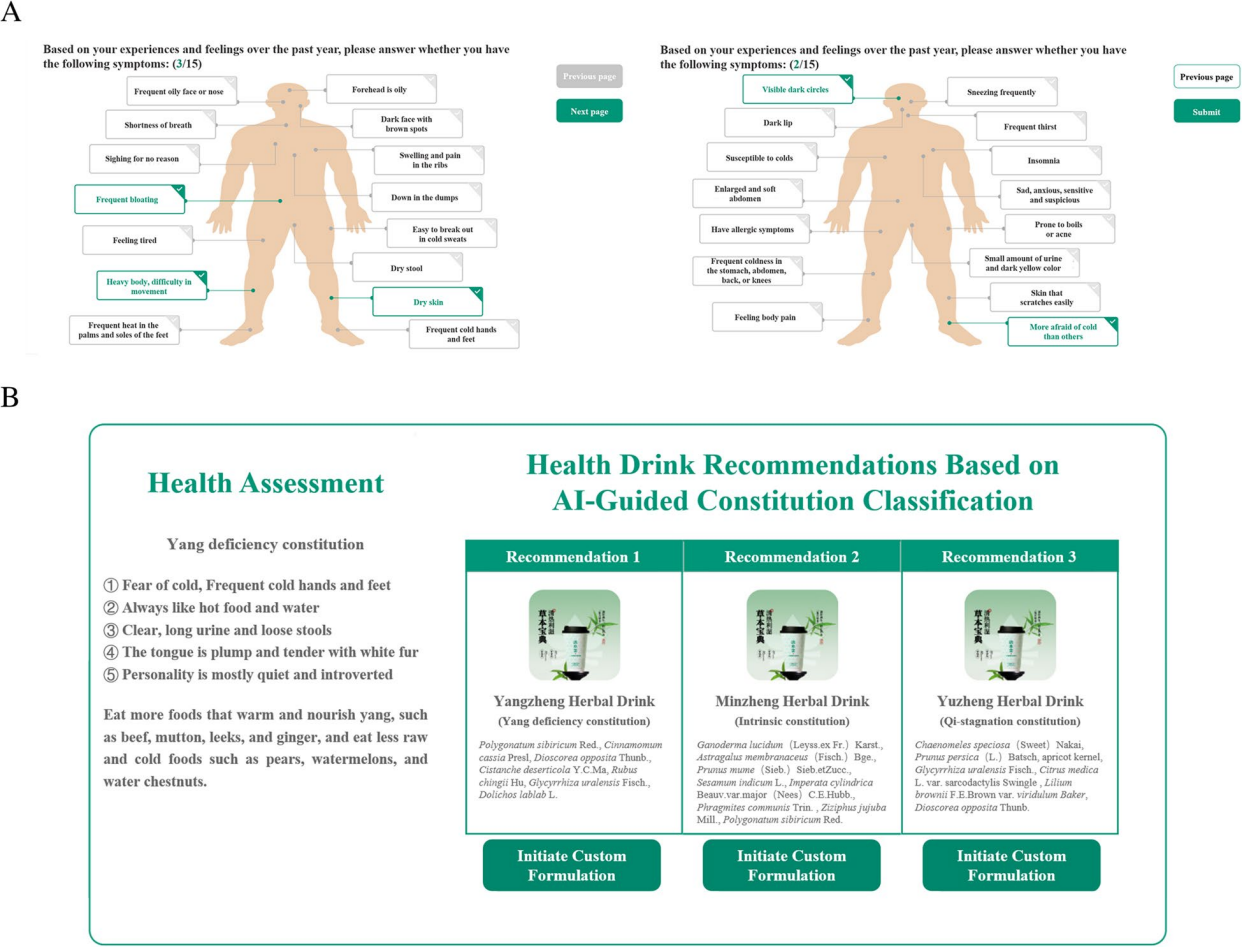
Interface and detailed information on the TCM-DS platform

Based on the TCM-DS model, we’ve developed a user-friendly platform for edible herbal formula recommendation (Fig. 4). The platform features an intuitive interface design where users can select their symptoms through single or multiple choices on the initial interface. Upon clicking the “submit” button, the system automatically analyzes the user’s constitution based on the structured questionnaire (displayed on the left panel) and recommends three suitable formulas (ordered by priority from left to right on the right panel). Although the system determines a single optimal recommendation through comprehensive analysis of both constitution and symptoms, it retains all three recommended formulas with their corresponding compatible constitution to preserve user choice.

Figure 4 presents a simulated case example. When a user reported symptoms including visible dark circles, more afraid of cold than others, frequent bloating, heavy body, difficult in movement, and dry skin (Fig. 4a), the system first identified the user as having a yang-deficient constitution (Fig. 4b). It then recommends yangzheng herbal drink as the optimal solution while providing minzheng herbal drink and yusheng herbal drink as alternative options (Fig. 4b). This design maintains scientific rigor in recommendations while ensuring practical flexibility in application.

## Discussion

In this study, we compiled a comprehensive dataset of TCM edible herbal formulas, which includes detailed information on symptoms, constitutions, formulas and their corresponding ingredients. By utilizing this dataset, we employed LoRA fine-tuning and RAG module to train a general-purpose LLM, ultimately developing a domain-specific model called TCM-DS. It achieves significantly superior performance in edible herbal formula recommendation task than existing general-purpose LLMs. Based on TCM-DS model, we developed an intelligent recommendation platform which incorporates a pipeline for TCM constitution analysis and personalized edible herbal formula recommendation. To our knowledge, TCM-DS represents the first domain-specific LLM designed for the application of *medicine and food homology* theory and is the first to integrate constitution theory with intelligent prescription recommendation systems.

Prior DeepSeek-based research in the TCM/MFH domain predominantly employed knowledge-graph–centric grounding for dietary advice or general retrieval and QA. For example, Yaoshi-RAG (DeepSeek-R1 combined with an uncertain KG and RAG) targeted MFH dietary recommendations [20], while OpenTCM (GraphRAG over classical corpora with DeepSeek-style LLMs) focused on knowledge retrieval and diagnostic QA [21]. Hospital-scale deployments of DeepSeek-R1 further support the feasibility of on-premises, privacy-preserving LLMs [22]. In this study, we propose TCM-DS model that specifically addressed edible herbal formula recommendation and explicitly incorporated constitution as a primary decision variable, which operationalize the clinical reasoning process from symptoms to syndrome/constitution to treatment principle to formula selection. Specifically, LoRA raises precision from 0.3848 to 0.6711, RAG alone reaches 0.9082, and LoRA plus RAG achieves 0.9924 (Table 1), with robustness to forward and reverse symptom orders (0.9924/0.9702, Table 2). To our knowledge, this is the first MFH-grounded, constitution-aware LLM dedicated to edible herbal formula recommendation, delivering prioritized, evidence-backed outputs rather than KG-augmented dietary advice or open-ended QA.

Although the superior performance of LLMs, techniques such as fine-tuning and RAG offer promising avenues for further enhancing their accuracy and relevance. Domain-specific fine-tuning enables LLMs to better understand and generate specialized content, thereby reducing hallucinations [12, 30]. In this study, we employed LoRA fine-tuning to the DeepSeek base model using a customized edible herbal formula dataset. Ablation experiments demonstrated significant performance improvement in recommendation task (Table 2). LoRA approach updates only the gradient of inserted low-rank matrices, substantially reducing both trainable parameters and computational costs. Similar approaches have shown effectiveness in related works. Yang et al.[11] employed LoRA to develop TCM-GPT-7B, demonstrating strong performance in TCM examination and TCM diagnosis. Pu et al. [18] alternatively incorporated TCM knowledge through attention mask matrix for prescription generation.

RAG module enables customization by dynamically integrating relevant external information. Functioning similarly to a search engine, RAG retrieves relevant and customized knowledge data in response to queries while enabling real-time updates. Its flexibility to plug into any LLM allows user integration with various base LLM and facilitates model updates. In healthcare applications, RAG-based LLMs incorporating clinical guidelines showed potential in clinical decision-making [31, 32]. Thyro-GenAI, an RAG-based chatbot, achieved clinician-level performance when generating responses for personalized clinical questions about thyroid disease with hallucination rates [32]. The GPT4 LLM-RAG model with international guideline produced more consistent output than humans with no hallucinations [33]. Compared to fine-tuning, RAG provides a more efficient solution without the need for extensive training examples or time. It achieved superior performance when injecting knowledge into LLMs in terms of data volume, accuracy, cost, time efficiency, and flexibility [34]. Similarly, our study also indicated greater performance enhancement by integrating RAG model as compared with fine-tuning (0.9082 vs 0.6711, Table 1). However, rather than being alternative approaches, RAG and fine-tuning serve as complementary techniques with their combination offering potential for further enhancement.

Additionally, this study innovatively incorporated Qi Wang’s constitution theory [24] into prescription recommendation system, representing a significant advancement beyond previous symptom-only recommendation models. By integrating TCM constitutions, we have developed a truly personalized TCM edible herbal formula recommendation model that better adheres to the TCM principle of *different treatments for the same disease*. For instance, for symptom clusters including dyspepsia, dampness stagnancy due to spleen deficiency, abdominal bloating, abdominal obesity, scanty dark urine, and fat, our proposed model provides personalized recommendations based on users’ TCM constitution: recommending the *Poria cocos appetizing drink* for Yin-deficiency constitution and the *dried tangerine peel fat loss drink* for phlegm-dampness constitution (Table S1).

In real-world applications, variability in symptom descriptions often creates mismatches with training data, thereby constraining model generalizability. In this study, we systematically evaluated model performance with both forward and reverse symptom sequences. Results showed that TCM-DS model exhibited optimal recommendation accuracy regardless of symptom sequence (Table 2), highlighting strong robustness. Notably, the non-adapted DeepSeek model achieved suboptimal performance, confirming its exceptional language understanding and generation capabilities, which empirically supports our choice of this base model.

There are several challenges that remain to be addressed. TCM-DS model primary rely on structured questionnaire inputs, which may constrain its practical application due to the highly heterogeneous symptom descriptions in real-world environments. Future work will focus on developing natural language processing module for unstructured symptom descriptions. We will also systematically incorporate key diagnostic dimension from TCM practice, including facial, tongue, and ocular diagnostics, to establish a more comprehensive diagnostic and recommendation model. Furthermore, continuous expansion of the symptom-constitution-formula database will be conducted to further improve model performance and clinical utility.

## Conclusion

This study presents TCM-DS, an intelligent model for personalized recommendation of TCM edible herbal formulas. By integrating domain-specific knowledge through LoRA fine-tuning and a RAG module, TCM-DS demonstrated superior performance compared to general-purpose LLMs. An intelligent recommendation platform developed based on TCM-DS model enable automated TCM constitution identification and generates personalized edible herbal drinks, offering novel insights for intelligent applications in TCM practice.

## Supplementary Information

Additional file 1

## Data Availability

The datasets used and/or analyzed during the current study are available from the corresponding author on reasonable request.

## Abbreviations

TCM: Traditional Chinese medicine
LLM: Large language model
NLP: Natural language processing
NHC: National Health Commission
SAMR: State Administration for Market Regulation
LoRA: Low-Rank Adaptation
RAG: Retrieval-Augmented Generation
