# Supplementary material for: TCM-DS: a large language model for intelligent traditional Chinese medicine edible herbal formulas recommendations
Source: Chin Med. 2025 Nov 17;20:191. doi: 10.1186/s13020-025-01249-0 (PMC12621383; doi:10.1186/s13020-025-01249-0)
Supplement: Supplementary file 1 — Additional file 1 [file 13020_2025_1249_MOESM1_ESM.docx]

Table S1. Samples in the edible herbal formula dataset.

| Sample 1 |  |
| --- | --- |
| Name | Dried tangerine peel fat loss drink  Chinese: 陈皮刮油饮 |
| Ingredients | *Citrus reticulata* Blanco, *Crataegus pinnatifida* Bge., *Nelumbo nucifera* Gaertn., *Lonicera japonica* Thunb., *Glycyrrhiza uralensis* Fisch., and Black tea  Chinese: 橘皮、山楂、荷叶、金银花、甘草、红茶 |
| Constitutions | Phlegm-dampness constitution  Chinese: 痰湿体质 |
| Symptoms | Dyspepsia, spleen deficiency with dampness exuberance, phlegm heat with cough, abdominal distension, abdominal obesity, scanty dark urine, and obesity  Chinese: 消化不良、脾虚湿盛、痰热咳嗽、腹胀、腹大肥软、小便短赤、肥胖 |
| Therapeutic effects | Regulate qi and fortify the spleen, clear heat and dry dampness, resolve phlegm, and lower lipid  Chinese: 理气健脾、清热燥湿、化痰、降脂 |
| Sample 2 |  |
| Name | Poria cocos appetizing drink  Chinese: 茯苓开胃饮 |
| Ingredients | *Crataegus pinnatifida* Bge., *Poria cocos* (Schw.) Wolf , *Citrus reticulata* Blanco, *Dioscotea opposita* Thunb., *Ziziphus jujuba* Mill., *Callus gallus domesticus* Brisson, and *Hordeum vulgare* L., isomaltooligosaccharide  Chinese: 山楂、茯苓、橘皮、山药、大枣、鸡内金、麦芽、低聚异麦芽糖 |
| Constitutions | Yin deficiency constitution  Chinese: 阴虚体质 |
| Symptoms | Dyspepsia, spleen deficiency with dampness exuberance, phlegm heat with cough, abdominal distension, abdominal obesity, scanty dark urine, and obesity  Chinese: 消化不良、脾虚湿盛、痰热咳嗽、腹胀、腹大肥软、小便短赤、肥胖 |
| Therapeutic effects | Fortify the spleen and promote digestion, and increase the appetite  Chinese: 健脾消食、开胃 |
| Sample 3 |  |
| Name | Bird's-nest and Collagen peptide beauty drink  Chinese: 燕窝胶原红颜饮 |
| Ingredients | Bird’s-nest, collagen peptide, *Malpighia emarginata* DC., resistant dextrin, *Citrus × sinensis* (L.) Osbeck, inulin, complex vitamins, *Aronia melanocarpa* (Michx.) Elliott, carrot  Chinese: 燕窝、胶原蛋白肽、针叶樱桃、抗性糊精、甜橙、菊粉、复配维生素、黑果花楸、胡萝卜 |
| Constitutions | Blood stasis constitution  Chinese: 血瘀体质 |
| Symptoms | Darkish complexion, dark circles, darkish complexion with chloasma, and lip hyperpigmentation  Chinese: 肤暗无光、黑眼圈、面暗褐斑、口唇暗 |
| Therapeutic effects | Tonify the collagen and nourish the skin  Chinese: 补充胶原蛋白、美颜养肤 |
| Sample 4 |  |
| Name | Ginseng and Longan warm drink  Chinese: 人参桂圆暖宫饮 |
| Ingredients | *Zingiber officinale* Rosc., *Citrus reticulata* Blanco, *Cinnamomum cassia* Presl, *Foeniculum vulgare* Mill., *Prunus persica* (L.) Batsch, *Ziziphus jujuba* Mill., *Glycyrrhiza uralensis* Fisch., brown sugar, *Dimocarpus longan* Lour., *Alpinia of ficinarum* Hance, *Panaxginseng* C.A.Mey.  Chinese: 干姜、橘皮、肉桂、小茴香、桃仁、大枣、甘草、红糖、桂圆、高良姜、人参 |
| Constitutions | Yang deficiency constitution  Chinese: 阳虚体质 |
| Symptoms | Qi deficiency with fatigable, blood deficiency with dysmenorrhea, reversal cold of the extremities, cold in the waist and back and knee, fear of cold, and fatigue  Chinese: 气虚易疲、血虚痛经、手脚冰凉、腰背膝凉、不耐寒冷、疲乏 |
| Therapeutic effects | Dissipate cold and warm the meridian，fortify the spleen and harmonize the stomach，move qi and replenish qi, and activate blood and resolve stasis  Chinese: 祛寒温经、健脾和胃、行气益气、活血化瘀 |

Table S2. Samples of prompt-completion pairs used in this study.

| *Prompts* | *Completions* |
| --- | --- |
| If the patient has the following symptoms: dyspepsia, spleen deficiency with dampness exuberance, phlegm heat with cough, abdominal distension, abdominal obesity, scanty dark urine, obesity. Then recommend | Dried tangerine peel fat loss drink |
| If the patient has the following symptoms: dyspepsia, spleen deficiency with dampness exuberance, phlegm heat with cough, abdominal distension, abdominal obesity, scanty dark urine, obesity. Then recommend | Poria cocos appetizing drink |
| If the patient has the following symptoms: darkish complexion, dark circles, darkish complexion with chloasma, lip hyperpigmentation. Then recommend | Bird's-nest and Collagen peptide beauty drink |
| If the patient has the following symptoms: qi deficiency with fatigable, blood deficiency with dysmenorrhea, reversal cold of the extremities, cold in the waist and back and knee, fear of cold, fatigue. Then recommend | Ginseng and Longan warm drink |

Table S3. LoRA Parameter

| Parameter | Value / Setting | Description |
| --- | --- | --- |
| torch_dtype | torch.bfloat16 | Enables mixed precision to reduce memory usage |
| device_map | "auto" | Automatically distributes model across GPUs |
| task_type | "CAUSAL_LM" | Task type: causal language modeling |
| target_modules | ["q_proj", "v_proj", "down_proj"] | Key attention modules for LoRA injection |
| r (rank) | 8 | Rank of low-rank adaptation matrices |
| lora_alpha | 32 | Scaling factor for LoRA |
| lora_dropout | 0.01 | Dropout rate to prevent overfitting |
| bias | "none" | Do not train bias parameters |
| inference_mode | FALSE | Enable gradient computation during training |
| per_device_train_batch_size | 4 | Training batch size per GPU |
| gradient_accumulation_steps | 2 | Simulates larger effective batch size |
| learning_rate | 2.00E-05 | Learning rate within recommended LoRA range |
| num_train_epochs | 3 | Number of training epochs |
| fp16 | TRUE | Enables half-precision training |
| optimizer | "adamw_torch" | AdamW optimizer from PyTorch |
| logging_steps | 50 | Logging frequency |
| save_steps | 500 | Checkpoint saving interval |
| warmup_steps | 100 | Learning rate warm-up steps |
| report_to | "tensorboard" | Enables visualization with TensorBoard |

Table S4. Performance comparison between base models.

| Model | Precision |
| --- | --- |
| LLaMA2-8B | 0.3642 |
| ChatGLM3-6B | 0.3581 |
| Mistral-7B | 0.2872 |
| Qwen2.5-7b | 0.1512 |
| deepseek-LLM-7B | **0.3848** |
